# What does a Pacman eat? Macrophagy and necrophagy in a generalist predator (*Ceratophrys stolzmanni*)

**DOI:** 10.7717/peerj.6406

**Published:** 2019-02-21

**Authors:** Diana Székely, Fernando P. Gaona, Paul Székely, Dan Cogălniceanu

**Affiliations:** 1 Faculty of Natural and Agricultural Sciences, Ovidius University Constanța, Constanța, Romania; 2 Laboratory of Fish and Amphibian Ethology, Behavioural Biology Unit, FOCUS, University of Liège, Liège, Belgium; 3 Association Chelonia Romania, Bucharest, Romania; 4 Departamento de Ciencias Biológicas, EcoSs Lab, Universidad Técnica Particular de Loja, Loja, Ecuador

**Keywords:** Feeding ecology, Amphibian, Food-chain, Predator, Dry forest

## Abstract

We describe for the first time the feeding ecology of the Pacific horned frog (*Ceratophrys stolzmanni*), as inferred through gastrointestinal tract content analysis and behavioural observations in its natural habitat. Ingested prey in adults ranged from mites and various insects to frogs and snakes. Prey items predominantly consisted of gastropods, non-formicid hymenopterans, and centipedes. We found no relationship between the size of the predator and the prey ingested, in terms of prey size, volume or number of items ingested. Additional direct observations indicate that all post-metamorphic stages are voracious, preying on vertebrates and engaging in anurophagy, cannibalism, and even necrophagy. Our study sheds light on the feeding habits of one of the least known species of horned frog.

## Introduction

A critical first step in understanding the ecology of a predator is to have knowledge of its food preferences and foraging strategy. Data regarding the type of prey consumed improves our understanding of the feeding strategy of the species, and uncovers its position in the trophic network (Jiang & Morin, 2005). In many ecosystems, especially in the tropics, amphibians are the most abundant vertebrates (Stebbins & Cohen, 1995), so they can have an important impact on food-webs, both as predators and prey (Semlitsch, O’Donnell & Thompson, 2014).

Frogs and toads swallow their prey whole, so they are considered gape-limited predators (Wells, 2010). Therefore, the width of their head constitutes a good predictor of the size of prey they are able to ingest (Emerson, 1986). As a result, a tendency for larger individuals or animals with wider mouths to consume larger prey items exists both between differently sized individuals of the same species (Lima & Moreira, 1993; Wu et al., 2005), but to some extent also between different species (Toft, 1980; Lima & Magnusson, 1998; Isacch & Barg, 2002). Among anurans, one family that seems to benefit from a series of morphological features associated with macrophagy is the Ceratophryidae (Emerson, 1985). Species belonging to this family have a disproportionately large, robust skull relative to their body size (hence the name of Pacman frogs vernacularly used for *Ceratophrys* species), a reduced skull length associated with an increase in skull width (Fabrezi, 2006) and fangs (Fabrezi & Emerson, 2003; Fig. S1). The capacity to ingest very large prey allows these animals to exploit diverse dietary resources, which results in a diet that consists, along with diverse invertebrates, of reptiles (Chávez, Venegas & Lescano, 2011), small birds and mammals (Duellman & Lizana, 1994; Schalk et al., 2014), and a large proportion of amphibians (Hulse, 1978; Pueta & Perotti, 2013; Stănescu, Marangoni & Reinko, 2014).

Among horned frogs, one species for which information regarding the feeding habits is lacking is the Pacific horned frog, *Ceratophrys stolzmanni*, a burrowing amphibian known from just a few locations in the coastal dry forests in Peru and Ecuador (IUCN Amphibian Specialist Group, 2018). The species is fossorial and nocturnal. Individuals remain buried in the ground most of their lives, and come out of their burrows only after heavy rains. Owing to its infrequent activity and small distribution, few studies concerning the ecology of the species have been carried out (Székely et al., 2017, 2018b). Pacific horned frogs inhabit seasonal environments, with a dry season consisting of at least 4 months with less than 10 mm monthly precipitation (Espinosa et al., 2016). The frogs aestivate during this interval, and return to activity and breed immediately after the first heavy rains of the rainy season (Székely et al., 2018a). However, even during the rainy season, they are essentially active above ground only during humid nights. First juveniles start to metamorphose at the end of March through April, depending on the precipitation regime; they have less than 3 months, generally until the end of June, to feed and grow enough that they can survive the subsequent aestivation. A previous study revealed that juveniles of this species double in size and reach sexual maturity less than a year after metamorphosis (Székely et al., 2018b), despite the short season in which they are active.

We describe here the first observations of the diet of the Pacific horned frog, obtained during the rainy season (January–April) of 2 years (2015–2016) in the natural habitat of the species. Additionally to reporting the variety of prey consumed, we also investigated the way the size of the predator influenced its diet; we hypothesize that individuals with wider heads would ingest larger prey (Quiroga, Sanabria & Acosta, 2009). We also expected a positive correlation between predator size and its ingested food in terms of either number of items or volume of food (Basso, 1988; Biavati, Wiederhecker & Colli, 2004), and that larger individuals would tend to avoid eating small items (Whitfield & Donnelly, 2006).

## Materials and Methods

### Ethics statement

This study was carried out in strict accordance with the guidelines for use of live amphibians and reptiles in field research compiled by the American Society of Ichthyologists and Herpetologists, The Herpetologists’ League and the Society for the Study of Amphibians and Reptiles. The research permit was issued by Ministerio del Ambiente del Ecuador (MAE-DNB-CM-2015-0016). This study was evaluated and approved by the Ethics Committee of Universidad Técnica Particular de Loja (UTPL-CBEA-2016-001).

### Study site

The study was conducted in Arenillas Ecological Reserve, El Oro Province, south-western Ecuador (03°34′S; 80°08′E, 30 m a.s.l.). The Arenillas Reserve is a lowland tropical dry forest, with a relatively stable climate in terms of temperature (mean annual temperature of 25 °C, with a 3.4 °C variation between the coldest and warmest months), but markedly seasonal in rainfall, with precipitation almost exclusively restricted to the 4 months of the wet season (January–April; on average, 515 mm of the 676 mm annual precipitation). The dry season has at least 4 months with less than 10 mm of monthly precipitation (Espinosa et al., 2016). A total of 10 species of amphibians are reported from the reserve (Székely et al., 2016), one of them being *C. stolzmanni.*

Fieldwork was conducted during the rainy season (January–April) in 2015 (38 days) and 2016 (65 days). Because of the conservation status of the species (Vulnerable according to IUCN Amphibian Specialist Group, 2018), we chose to use only non-invasive methods: the gastrointestinal tract (GIT) content analysis of animals found dead and the direct observation of feeding behaviour of the animals in their natural habitat.

**Gastrointestinal tract (GIT) content analysis.** The analysis of GI tract contents was performed on 14 road-kills and 23 individuals found dead (presumably drowned) at the reproduction ponds, after mass mating events. In horned frogs, reproductive events are sudden and brief, lasting only one night (Székely et al., 2018a), and individuals continue feeding even while in amplexus (Schalk & Montaña, 2011; Schalk et al., 2014). We only used freshly killed animals with intact abdominal cavities. To minimize the effect of digestion, individuals were dissected within 2 h of collection. All contents of the whole digestive system, from mouth to any undigested items encountered in the hindgut, were stored in plastic tubes filled with 96% alcohol until examination. Since juveniles are extremely delicate, they were severely damaged by the impact with vehicles. Therefore, no juveniles could be used in our study, and the sample consisted exclusively of adults. For each individual we recorded the sex, males being recognized by the presence of secondary sexual characters (i.e. dark coloration on the throat and nuptial pads on forelimbs; Ortiz, 2018). Depending on the state of the frog, we also measured the snout-vent length (SVL) and head width (HW) with a Dial-Max calliper (0.1 mm precision), and body mass (BM) with My Weigh Triton T3 portable scale (0.01 g precision). Specimens that were suitable for preservation were deposited at the Museo de Zoología de la Pontificia Universidad Católica del Ecuador, Quito, Ecuador and the Museo de Zoología, Universidad Técnica Particular de Loja, Loja, Ecuador (Table S1).

Gastrointestinal tract contents were analysed in the lab, using an Olympus SZ61 Stereo Microscope with an ocular micrometre attached. Prey items were counted and identified to the lowest taxonomic group possible, usually order. The abundance of a particular prey type (*n_x_%*) was calculated as the percentage of encountered items of that type from the total number of prey items, while the frequency of occurrence (*f_x_*) was the percentage of animals that contained the particular type of prey from the total number of animals that contained food in their GIT. For each intact prey item, the maximum length and width were measured to the nearest 0.1 mm. For semi-digested or incomplete prey, we estimated length and width based on their intact parts (e.g. body, legs). For vertebrate prey, accurate estimation of volume was not possible, so they were excluded from further analysis. Estimation of the volume of each prey item was done using the formula for a prolate spheroid (Colli & Zamboni, 1999; Vitt, Zani & Caldwell, 2009):

$$v = {4 \over 3}{\rm{\pi }}\left( {{{{\rm{length}}} \over {\rm{2}}}} \right){\left( {{{{\rm{width}}} \over {\rm{2}}}} \right)^2}$$

and afterwards volumetric percentages were calculated for each prey type out of the total volume of all prey (*v_x_*%). The relative importance index of a particular prey type (*I_x_*, indicating the relative importance of a specific category of prey in the diet) was estimated using the formula of Biavati, Wiederhecker & Colli (2004): *I_x_* = (*n_x_*% + *f_x_*%+ *v_x_*%)/3.

The existence of relationships between predator size and prey number, size and volume was investigated using Pearson correlations. We tested for significant correlations between the body length (SVL) of the frog on the one hand, and the number of prey items, the minimum volume of individual prey item, or the total ingested volume, on the other. We also tested for correlations between predator HW and the maximum prey width it ingested. Analyses were carried out with SPSS software (version 21.0; IBM Corp., Armonk, NY, USA), with a significance level of 0.05. Values are reported as mean ± S.E.

**Direct observation of feeding behaviour.** Field surveys in the study area were carried out during both day and night. Whenever we encountered horned frogs in the process of feeding, we obtained as much information as possible without disturbing the animal; manipulation was avoided, and animals were photographed from above, alongside a reference object. Based on these photographs, their SVL was estimated using ImageJ 1.46r software (Schneider, Rasband & Eliceiri, 2012). However, if during the observation the frog released its prey, both predator and prey were captured, their SVLs were measured using dial callipers, and they were then released.

## Results

**Figure 1:**
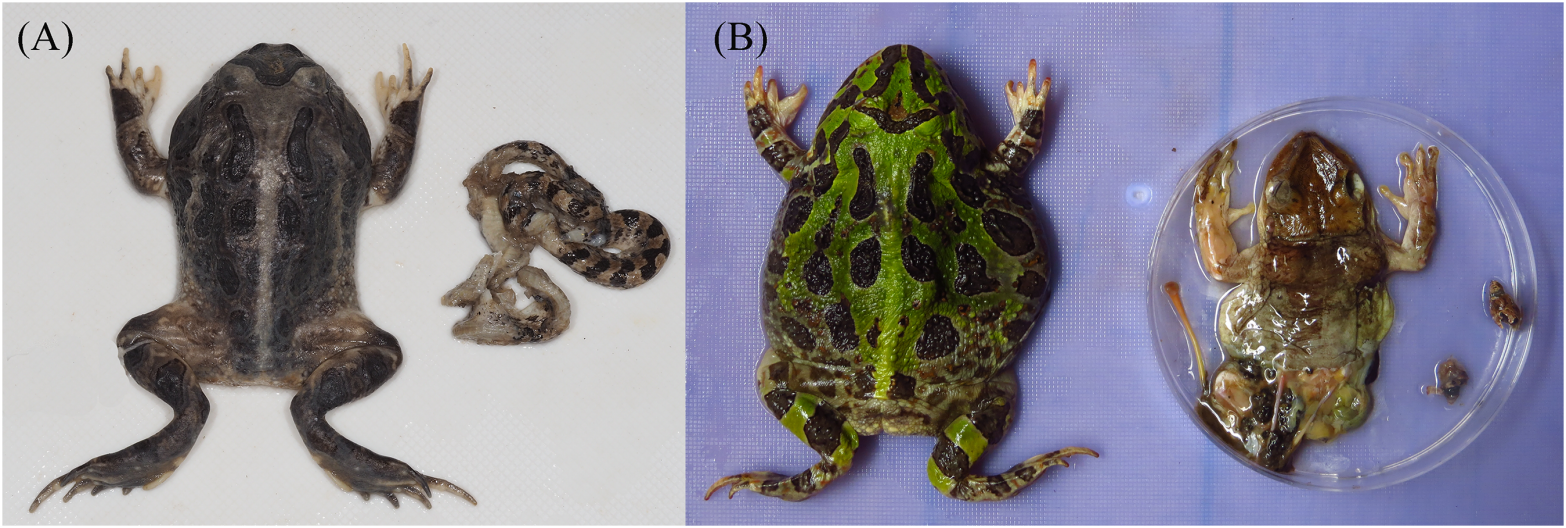
Vertebrate prey of *Ceratophrys stolzmanni* from Arenillas Ecological Reserve, Ecuador; each image shows the predator on the left and its prey on the right. (A) Northern Cat-eyed snake (*Leptodeira septentrionalis*); (B) Jordan’s Casque-headed Treefrog (*Trachycephalus jordani*). Photo credit: Diana Székely.

**GIT content analysis.** From the 37 collected dead horned frogs (19 females, 18 males, SVL ranging between 51.6 and 81.2 mm), six (16%) had no detectable food in their GIT. From the remaining 31 frogs, we retrieved 197 prey items, belonging to 12 taxa (Table 1), including three vertebrate items (two anurans, *Trachycephalus jordani* and *Leptodactylus labrosus*, and a juvenile snake, *Leptodeira septentrionalis*; Fig. 1). The average number of prey items per individual was 6.4 ± 3.8, ranging between 1 and 121.

**Table 1:** Prey types present in the gastrointestinal tract of adult (*n* = 31) *Ceratophrys stolzmanni* from Arenillas Ecological Reserve.

Prey taxa	*n_x_* (a)	*n_x_* (%)	*f_x_* (a)	*f_x_* (%)	*V_x_* (a)	*V_x_* (%)	*I_x_*
Pulmonata	19	9.6	14	45.2	10151.1	33.3	29.4
Arachnida	126	64	7	22.6	383.4	1.2	29.3
Acari	120	60.9	1	3.2	0.5	0	21.4
Araneae	5	2.5	5	16.1	306.2	1	6.6
Pseudoscorpionida	1	0.5	1	3.2	14.4	0.1	1.3
Chilopoda	2	1.0	2	6.5	8,990.9	29.5	12.3
Insecta	47	23.4	15	48.4	10,946.4	35.9	35.9
Coleoptera	8	4.1	5	16.1	4,513.2	14.8	11.7
Hymenoptera F	13	6.6	4	12.9	96.8	0.3	6.6
Hymenoptera NF	23	11.7	8	25.8	1,641	5.4	14.3
Lepidoptera	1	0.5	1	3.2	261.7	0.9	1.5
Orthoptera	1	0.5	1	3.2	4,421.1	14.5	6.1
Unidentified	1	0.5	1	3.2	12.6	0.04	1.3
Anura	2	1.0	2	6.5	n.a.	n.a.	n.a.
Serpentes	1	0.5	1	3.2	n.a.	n.a.	n.a.

**Note:**

*n_x_*, abundance (number of prey items); *f*_x_, frequency (number of individuals where a type of prey was encountered); *v_x_*, volume (in mm^3^), given in absolute (a) and percentage (%) values. *I_x_*, index of relative importance. n.a., volume and importance index could not be estimated. Hymenoptera F, Formicidae; Hymenoptera NF, non-formicid hymenopterans.

**Figure 2:**
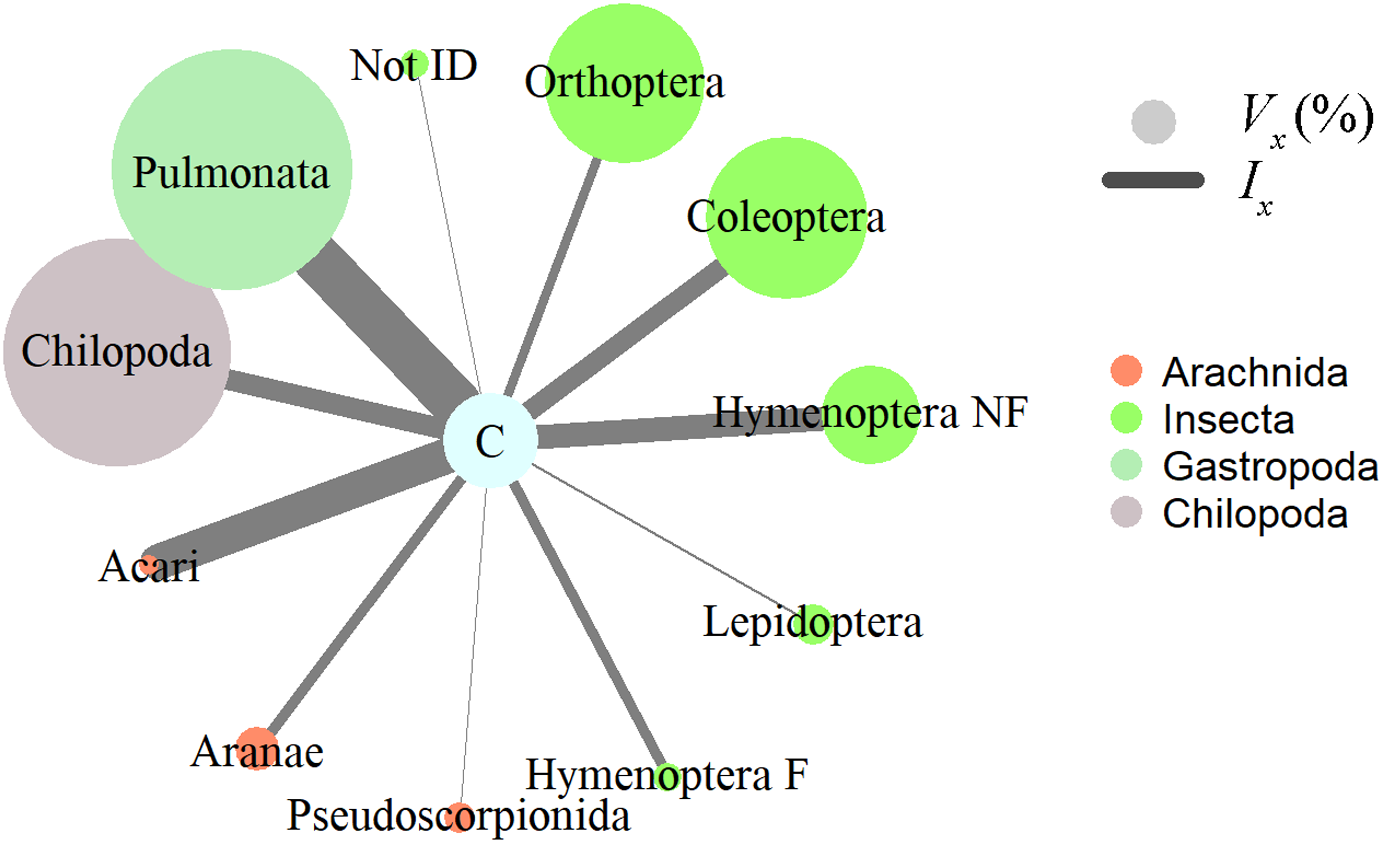
Invertebrate prey items relative importance in the diet of *Ceratophrys stolzmanni* (*n* = 31), as deduced from their gastrointestinal tract contents. *V_x_* (%), relative volume representation; *I*_x_, index of relative importance. Hymenoptera F, Formicidae; Hymenoptera NF, non-formicid hymenopterans. Vertebrate prey encountered were not included in the analysis, due to the advanced state of digestion.

Insects, gastropods, and arachnids represented the most important prey as revealed by the relative importance index; in terms of volume, gastropods (Pulmonata) and centipedes (Chilopoda) represented a large proportion, while numerically mites (Acari) and non-ant Hymenoptera dominated the diet of horned frogs (Fig. 2). Ingested invertebrate prey varied in length between 1 and 65 mm (mean ± S.E. = 13.2 ± 1.6 mm) and in width between 1 and 16 mm (5.6 ± 0.6 mm). In the 31 frogs that had prey items, we found no statistically significant relationship between the predator size and the ingested prey size. The total length (SVL) of the predator was not correlated with the number of prey items it contained (Pearson correlation; *r* = −0.108, *p* = 0.564, *n* = 31), the total volume of GIT contents (Pearson correlation; *r* = 0.197, *p* = 0.345, *n* = 25), or the minimum volume of any individual ingested item (Pearson correlation; *r* = −0.005, *p* = 0.981, *n* = 25). The HW was not correlated with the maximum width of ingested prey (Pearson correlation; *r* = 0.192, *p* = 0.357, *n* = 25).

**Figure 3:**
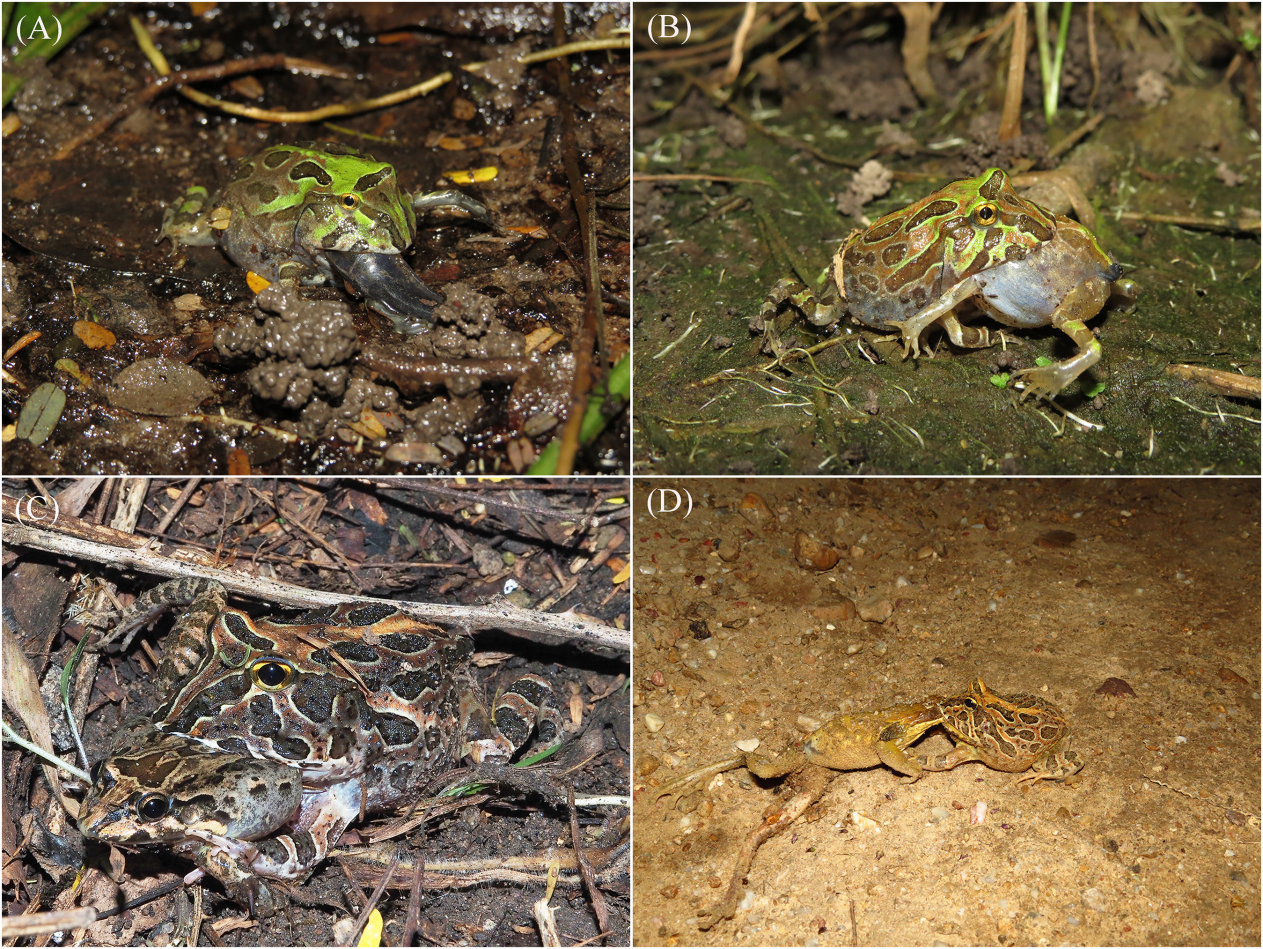
Anurophagy in *Ceratophrys stolzmanni.* (A) and (B) Cannibalism in juveniles; (C) adult *C. stolzmanni* preying upon a *Leptodactylus labrosus*; (D) adult *C. stolzmanni* scavenging on a *Trachycephaus jordanni* carcass. Photo credit: Diana Székely.

**Direct observation of feeding behaviour.** Observed behaviours included both predation and scavenging on anuran species (Fig. 3). We witnessed 22 instances of juveniles (Gosner stage 46, tail completely resorbed, average SVL = 29.6 ± 1.3 mm) performing cannibalistic attacks on similar or younger froglets (tail in various stages of resorption, SVL = 23.8 ± 1.2 mm, *n* = 18, the rest being too far ingested to estimate the size). The average ratio between predator and prey SVL was 1.26 (Table S2). The prey was always caught head-first. In one additional case, we did not witness the predation event, but the dorsal pattern of a Pacific horned frog was evident by transparency through the stretched skin of the cannibal juvenile’s abdomen.

We encountered five instances of adult *C. stolzmanni* (mean SVL = 55.9 ± 2.4 mm) feeding on *Leptodactylus labrosus* (45.7 ± 3.2 mm, *n* = 4, one escaped before it could be photographed or measured). The average ratio between predator and prey SVL was 1.24 (Table S2). The prey was bitten by the posterior part, and the horned frogs used their forelimbs to subdue it and secure a better position for ingestion (Fig. 3C). As a defence mechanism, the white-lipped frogs were inflated and emitted a high-pitched distress call.

**Necrophagy.** On March 19, 2015 at around 23:00 on a dirt road inside the reserve, we encountered a male *C. stolzmanni* (SVL = 58 mm), attempting to ingest a male *T. jordani* (SVL = 73 mm) that was dead on the road (Fig. 3D). The condition of the carcass, which was flattened and partially dried out, indicated that the road-killed treefrog had been dead for several hours. We observed the pair for about 10 min. During this interval, the horned frog, having bitten the treefrog by the head, jumped and used its forelegs to attempt to position its evidently larger prey for an easier ingestion (Video S1). Since the horned frog was noticeably disturbed by our presence and tried to hide under the vegetation by the side of the road, we left. We cannot know whether the feeding attempt was successful.

## Discussion

The few studies concerning the dietary habits of *Ceratophrys* species in their natural habitat reveal them as generalist and opportunistic predators (Pueta & Perotti, 2013; Schalk et al., 2014). Our study shows that, similar to its larger congeners, the Pacific horned frogs hunt and consume diverse resources. Generalist feeders, switching between invertebrates and vertebrates, have more feeding opportunities and thus higher growth rates compared to more specialized competitors (Caley & Munday, 2003; Berumen & Pratchett, 2008).

For other species of this genus, vertebrate prey represent a predominant part of their diet. The more in-depth studies concerning the diet of *C. cornuta* (Duellman & Lizana, 1994), *C. joazeirensis* (Da Silva Jorge et al., 2015) and *C. ornata* (Basso, 1988) show that vertebrates represent a high volumetric (up to 98%) and numeric (up to 69%) proportion of the their food. In the case of the Pacific horned frog, only approximately 10% of the individuals had preyed upon amphibians and reptiles, and vertebrates represented 1.5% of all ingested prey items. However, although the advanced state of digestion did not allow us to accurately estimate their volume, they constitute a large part of the total consumed volume. The additional direct observations of anurophagy confirm that such events are not accidental. Other vertebrates, such as rodents and birds, which were reported from the diet of large *Ceratophrys* species such as *C. cornuta* (Duellman & Lizana, 1994), *C. cranwelli* (Schalk et al., 2014) and *C. ornata* (Basso, 1988), are less likely to be feasible to the smaller sized Pacific horned frog. The differences in the amount of vertebrate prey consumed are likely a consequence of the smaller size of *C. stolzmanni* as well.

In amphibians, the width of the mouth limits the size of items that can be ingested (Emerson, 1985). Indeed, a number of studies show that as individuals grow in size, their diet also shifts towards larger prey (Lima & Moreira, 1993; Hirai, 2002). As a result, we expected to find that larger Pacific horned frogs consumed larger prey. However, no relation between the predator gape and the maximum prey item ingested was evident in our study. The lack of correlation between predator and prey is probably due to the lack of juveniles in our sample. A study carried out across size classes would probably confirm that larger individuals have access to and consistently eat larger food items. On the other hand, our results show that even large individuals capture and consume very small prey items, such as mites and small insects. Eating small prey may be beneficial because of lower behavioural costs and risks (e.g. handling time and energy expenditure; risk of injury), even in larger adults (Simon & Toft, 1991).

In some aspects, the taxonomic composition of invertebrate prey in *C. stolzmanni* showed some differences compared to other horned frogs for which data is available. An important proportion of their diet consisted of molluscs (Gastropoda), which were consumed by almost half of the sampled frogs. This contrasts with the rare occurrence of gastropods in other *Ceratophrys* (Schalk et al., 2014). The dominance of ants (Formicidae), which was reported for *C. cornuta* (Duellman & Lizana, 1994) and *C. aurita* (Schalk et al., 2014), is also not shared by the Pacific horned frogs, as ants did not represent a substantial part of their diet. Although amphibians are capable of distinguishing between prey characteristics, resulting in consistent selectivity of prey (Freed, 1982), these differences in diet may simply reflect the discrepancies in resource availability rather than an active choice. Future studies focusing on diet vs. site-specific prey availability should clarify these questions (Cogălniceanu et al., 2018).

**Anurophagy and cannibalism.** The majority of anurans feed mostly on invertebrates (Pough et al., 2016), but some species occasionally consume post-metamorphic anurans (Toledo, Ribeiro & Haddad, 2007). This occurs with a much higher frequency in a few genera (Measey et al., 2015), such as *Lithobates* (Bolek & Janvy, 2004; Silva et al., 2009), *Leptodactylus* (Rodrigues et al., 2011; Costa-Pereira et al., 2015), and *Ceratophrys* (Schalk et al., 2014). At least two species of the genus *Ceratophrys* exhibit complex pedal luring behaviour (Murphy, 1976; Radcliffe et al., 1986), which presumably evolved to attract visually hunting prey, such as other anurans (Hagman & Shine, 2008; Sloggett & Zeilstra, 2008). Feeding on vertebrates is beneficial: they are a source of energy that is easier to digest compared to most groups of invertebrates, which have a tough exoskeleton (Secor, Wooten & Cox, 2007). Additionally, the higher protein of vertebrates promotes rapid growth (Grayson et al., 2005), while the high water content of anuran prey compared to invertebrates can be especially advantageous in a xeric environment.

Intra-guild predation can be considered as an opportunistic form of predation (Toledo, Ribeiro & Haddad, 2007). Additional to a phylogenetic predisposition (Measey et al., 2015), this behaviour is favoured by several environmental conditions, such as overlap in habitat use amongst various size classes (Wissinger et al., 2010), a scarcity in alternative prey (Pizzatto & Shine, 2008), and a high amphibian density (Polis & Myers, 1985). The conditions favouring cannibalism are met especially in highly seasonal environments (Blayneh, 1999). In the study area, shortly after metamorphosis, the density of *C. stolzmanni* juveniles in the vicinity of breeding ponds can be as high as 25 individuals/m^2^ (D. Székely, personal observation, 2016), and their capacity to ingest large prey increases the probability for cannibalism. Along with providing a nutritious source of food, consuming other anurans found in the same habitat can also be beneficial by eliminating potential competitors (Polis, Myers & Holt, 1989; Finke & Denno, 2003).

**Necrophagy.** Scavenging is common in some anuran species during their larval stage (Heinen & Abdella, 2005; Silva & Muniz, 2005; Kirchmeyer et al., 2015), since tadpoles base the recognition of food items on chemical cues and are typically grazing feeders (Altig, Whiles & Taylor, 2007). However, reports of adults consuming any type of carcass are exceptional (Beane & Pusser, 2005; Tupper, Adams & Timm, 2009; Oda & Landgraf, 2012). In post-metamorphic anurans, a certain pattern of movement is necessary for prey recognition and thus for triggering an attack (Ewert, Burghagen & Schurg-Pfeiffer, 1983), so that cadavers are generally disregarded. Even if scavenging is occasional in *C. stolzmanni*, our report suggests that some anuran species have greater dietary flexibility than often believed.

## Conclusions

We found that the Pacific horned frog has a relatively diverse diet, both in terms of prey diversity and size. The ability to feed on a wide variety of prey types indicates that the species has the capacity to take advantage of a variety of feeding opportunities. However, we acknowledge that the number of individuals used in this study is low and we are still far from having a complete understanding of this species’ feeding habits. Further research on the subject is needed. Additionally, studies testing consumed vs available resources and competitive interactions with other species would allow for conclusions regarding the selectivity (or lack thereof) in prey choice. Such studies would also permit testing for the active inclusion of vertebrates in their diet (Toft, 1981). A lack of selection in prey can have important conservation implications, suggesting that the species might be more resilient to shifts in community composition caused by habitat change (Raubenheimer, Simpson & Tait, 2012) or change in prey diversity (Lister & Garcia, 2018). Also, generalist feeders, especially higher-order consumers, can have a stabilizing effect on food-webs (Rooney et al., 2006). On the other hand, it can be speculated that species which frequently engage in intra-guild predation, cannibalism and necrophagy might be especially at risk in the case of an emerging disease or parasite, since such behaviour increases the risk of transmission of different diseases (Pfennig, 2000; Harp & Petranka, 2006; Brunner, Schock & Collins, 2007).

## Supplemental Information

10.7717/peerj.6406/supp-1 Supplemental Information 1 Fangs in *Ceratophrys stolzmanni*. (a) Ossified odontoids, projections from the lower jaw similar to fangs. (b) Monocuspid teeth. Photo credit: Diana Székely.

10.7717/peerj.6406/supp-2 Supplemental Information 2 Voucher numbers, collection date and locality for some of the specimens used in the study. MUTPL–Museo de Zoología, Universidad Técnica Particular de Loja, Loja, Ecuador; QCAZ–Museo de Zoología de la Pontificia Universidad Católica del Ecuador, Quito, Ecuador.

10.7717/peerj.6406/supp-3 Supplemental Information 3 Morphometric information for encountered *Ceratophrys stolzmanni* predating on anurans. SVL–snout-vent length (mm), n.a.–data not available, since prey was ingested too far for approximation.

10.7717/peerj.6406/supp-4 Supplemental Information 4 Necrophagy in the Pacific horned frog. Video credit: Diana Székely.

10.7717/peerj.6406/supp-5 Supplemental Information 5 Raw data.

## Additional Information and Declarations

### Field Study Permissions

The following information was supplied relating to field study approvals (i.e. approving body and any reference numbers): Permits for this study were issued by Ministerio del Ambiente de Ecuador (MAE-DNB-CM-2015-0016) granted to Universidad Técnica Particular de Loja (UTPL).

### Data Availability

The following information was supplied regarding data availability: The raw measurements are provided in the Table S1 (Morphometric information for encountered *Ceratophrys stolzmanni* predating on anurans). These data were used for the statistical analysis.
