# Supplementary figures and images for: What does a Pacman eat? Macrophagy and necrophagy in a generalist predator (Ceratophrys stolzmanni)
Source: PeerJ. 2019 Feb 21;7:e6406. doi: 10.7717/peerj.6406 (PMC6387761; doi:10.7717/peerj.6406)

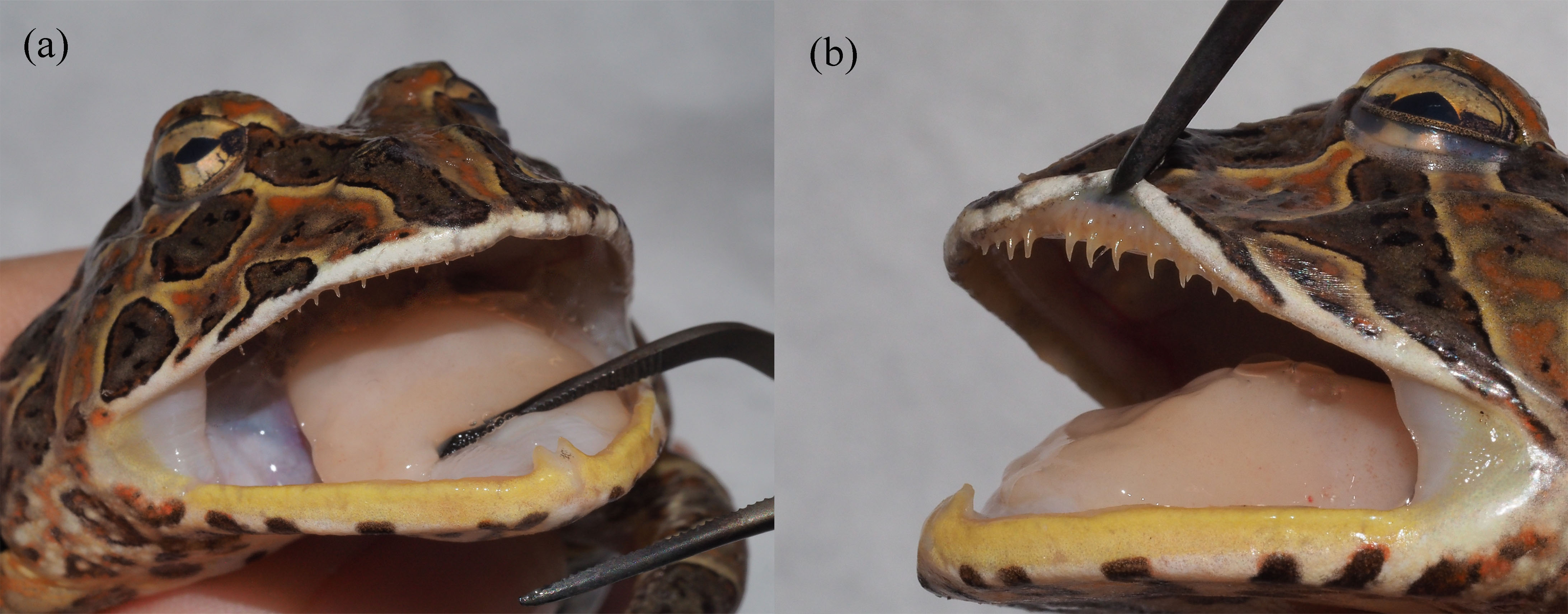

Supplement: Supplemental Information 1 — (a) Ossified odontoids, projections from the lower jaw similar to fangs. (b) Monocuspid teeth. Photo credit: Diana Székely. [file peerj-07-6406-s001.png]
