# Supplementary material for: What does a Pacman eat? Macrophagy and necrophagy in a generalist predator (Ceratophrys stolzmanni)
Source: PeerJ. 2019 Feb 21;7:e6406. doi: 10.7717/peerj.6406 (PMC6387761; doi:10.7717/peerj.6406)
Supplement: Supplemental Information 3 — SVL–snout-vent length (mm), n.a.–data not available, since prey was ingested too far for approximation. [file peerj-07-6406-s003.docx]

| **Predator SVL** | **Prey species** | **Estimated prey SVL** | **Ratio predator/prey SVL** |
| --- | --- | --- | --- |
| 60.4 | *Leptodactylus labrosus* | 51.1 | 1.18 |
| 52.2 | *Leptodactylus labrosus* | 44.3 | 1.18 |
| 62.5 | *Leptodactylus labrosus* | 50.1 | 1.25 |
| 54.4 | *Leptodactylus labrosus* | n.a. | n.a. |
| 50 | *Leptodactylus labrosus* | 37.4 | 1.34 |
| n.a. | *Ceratophrys stolzmanni* | n.a. | n.a. |
| 27.7 | *Ceratophrys stolzmanni* | 19 | 1.46 |
| n.a. | *Ceratophrys stolzmanni* | n.a. | n.a. |
| 33 | *Ceratophrys stolzmanni* | 26.2 | 1.26 |
| 37.9 | *Ceratophrys stolzmanni* | 33.1 | 1.15 |
| 28.6 | *Ceratophrys stolzmanni* | 25.7 | 1.11 |
| 23.3 | *Ceratophrys stolzmanni* | 20.7 | 1.12 |
| 21.6 | *Ceratophrys stolzmanni* | n.a. | n.a. |
| 26.2 | *Ceratophrys stolzmanni* | n.a. | n.a. |
| 28.8 | *Ceratophrys stolzmanni* | 20 | 1.44 |
| 25.4 | *Ceratophrys stolzmanni* | 18.9 | 1.34 |
| 41.8 | *Ceratophrys stolzmanni* | 33 | 1.27 |
| 30.3 | *Ceratophrys stolzmanni* | 22.1 | 1.37 |
| 40.3 | *Ceratophrys stolzmanni* | 33.2 | 1.21 |
| 29 | *Ceratophrys stolzmanni* | 25.3 | 1.15 |
| 23 | *Ceratophrys stolzmanni* | 18.1 | 1.27 |
| 30.7 | *Ceratophrys stolzmanni* | 26.1 | 1.18 |
| 30.6 | *Ceratophrys stolzmanni* | 24.9 | 1.23 |
| 21.1 | *Ceratophrys stolzmanni* | 20.5 | 1.03 |
| 30.7 | *Ceratophrys stolzmanni* | 24.7 | 1.24 |
| 25.2 | *Ceratophrys stolzmanni* | 17.4 | 1.34 |
| 25.4 | *Ceratophrys stolzmanni* | 19 | 1.45 |
